# Structural variations in a non-coding region at 1q32.1 are responsible for the NYS7 locus in two large families

**DOI:** 10.1007/s00439-020-02156-0

**Published:** 2020-04-04

**Authors:** Wenmin Sun, Shiqiang Li, Xiaoyun Jia, Panfeng Wang, J. Fielding Hejtmancik, Xueshan Xiao, Qingjiong Zhang

**Affiliations:** 1 State Key Laboratory of Ophthalmology, Zhongshan Ophthalmic Center, Sun Yat-Sen University, 54 Xianlie Road, Guangzhou, 510060 China; 2 Ophthalmic Genetics and Visual Function Branch, National Eye Institute, National Institutes of Health, Bethesda, MD USA

## Abstract

Congenital motor nystagmus (CMN) is characterized by early-onset bilateral ocular oscillations without other ocular deficits. To date, mutations in only one gene have been identified to be responsible for CMN, i.e., *FRMD7* for X-linked CMN. Four loci for autosomal dominant CMN, including NYS7 (OMIM 614826), have been mapped but the causative genes have yet to be identified. NYS7 was mapped to 1q32.1 based on independent genome-wide linkage scan on two large families with CMN. In this study, mutations in all known protein-coding genes, both intronic sequence with predicted effect and coding sequence, in the linkage interval were excluded by whole-genome sequencing. Then, long-read genome sequencing based on the Nanopore platform was performed with a sample from each of the two families. Two deletions with an overlapping region of 775,699 bp, located in a region without any known protein-coding genes, were identified in the two families in the linkage region. The two deletions as well as their breakpoints were confirmed by Sanger sequencing and co-segregated with CMN in the two families. The 775,699 bp deleted region contains uncharacterized non-protein-coding expressed sequences and pseudogenes but no protein-coding genes. However, Hi-C data predicted that the deletions span two topologically associated domains and probably lead to a change in the 3D genomic architecture. These results provide novel evidence of a strong association between structural variations in non-coding genomic regions and human hereditary diseases like CMN with a potential mechanism involving changes in 3D genome architecture, which provides clues regarding the molecular pathogenicity of CMN.

**Electronic supplementary material:**

The online version of this article (10.1007/s00439-020-02156-0) contains supplementary material, which is available to authorized users.

## Introduction

Nystagmus is a condition characterized by involuntary oscillation of eyes. Infantile nystagmus, onset in the first 6 months of life, has been reported to occur in 1 of 821 live births (Nash et al. 2017). It is a common sign of some ocular diseases, including hereditary retinal diseases, albinism, aniridia, congenital cataract, etc. Congenital motor nystagmus (CMN), also termed as idiopathic infantile nystagmus, occurs without other ocular sensory deficits and is one of the most common types of infantile nystagmus, accounting for approximately 31.0% of the total cases (Nash et al. 2017). CMN is considered to result from malfunction of areas of the brain responsible for eye movement control since patients with CMN usually show visual acuity close to normal (Sarvananthan et al. 2009).

CMN can be transmitted as an autosomal dominant, autosomal recessive, or X-linked trait. To date, at least six loci for CMN have been identified by genome-wide linkage studies. Of the six loci, four are autosomal dominant, namely, NYS2 (OMIM 164100, 6p12) (Kerrison et al. 1996), NYS3 (OMIM 608345, 7p11.2) (Klein et al. 1998), NYS4 (OMIM 193003, 13q31–q33) (Ragge et al. 2003), and NYS7 (OMIM 614826, 1q31.3–q32.1) (Li et al. 2012; Xiao et al. 2012); while the other two are X-linked, that is, NYS1 (OMIM310700, Xq26.2) (Guo et al. 2006; Kerrison et al. 1999, 2001; Mellott et al. 1999) and NYS5 (OMIM 300589, Xp11.4) (Cabot et al. 1999). Of the six loci, mutations in *FRMD7* (OMIM 300628) have been shown to be responsible for NYS1, which might account for 22–35% of CMN (AlMoallem et al. 2015; Choi et al. 2018; Tarpey et al. 2006); while, genes responsible for the other five loci await identification.

We previously mapped NYS7 based on independent genome-wide linkage analyses on two large families with autosomal dominant CMN (Li et al. 2012; Xiao et al. 2012). Of the two families, whole-exome sequencing (WES) of one family and candidate gene analysis of the other failed to identify any potential pathogenic variants in genes situated inside the linkage interval.

In this study, whole-genome sequencing based on the HiSeq platform and long-read whole-genome sequencing based on the Nanopore platform were carried out on samples from the two families with CMN mapped to NYS7. Two different large fragment deletions, with an overlapping region of 775,699 bp not containing any known protein-coding genes, were identified in the shared linkage interval in the two families. It is interesting that the shared region of the deletions was predicted to span two topologically associated domains (TADs), probably leading to a change in 3D genome architecture. These results provide novel evidence of a strong association between structural variants (SVs) in genomic regions devoid of protein-coding genes and human hereditary disease possibly by changing the 3D genome architecture.

## Materials and methods

### Subjects

**Fig. 1:**
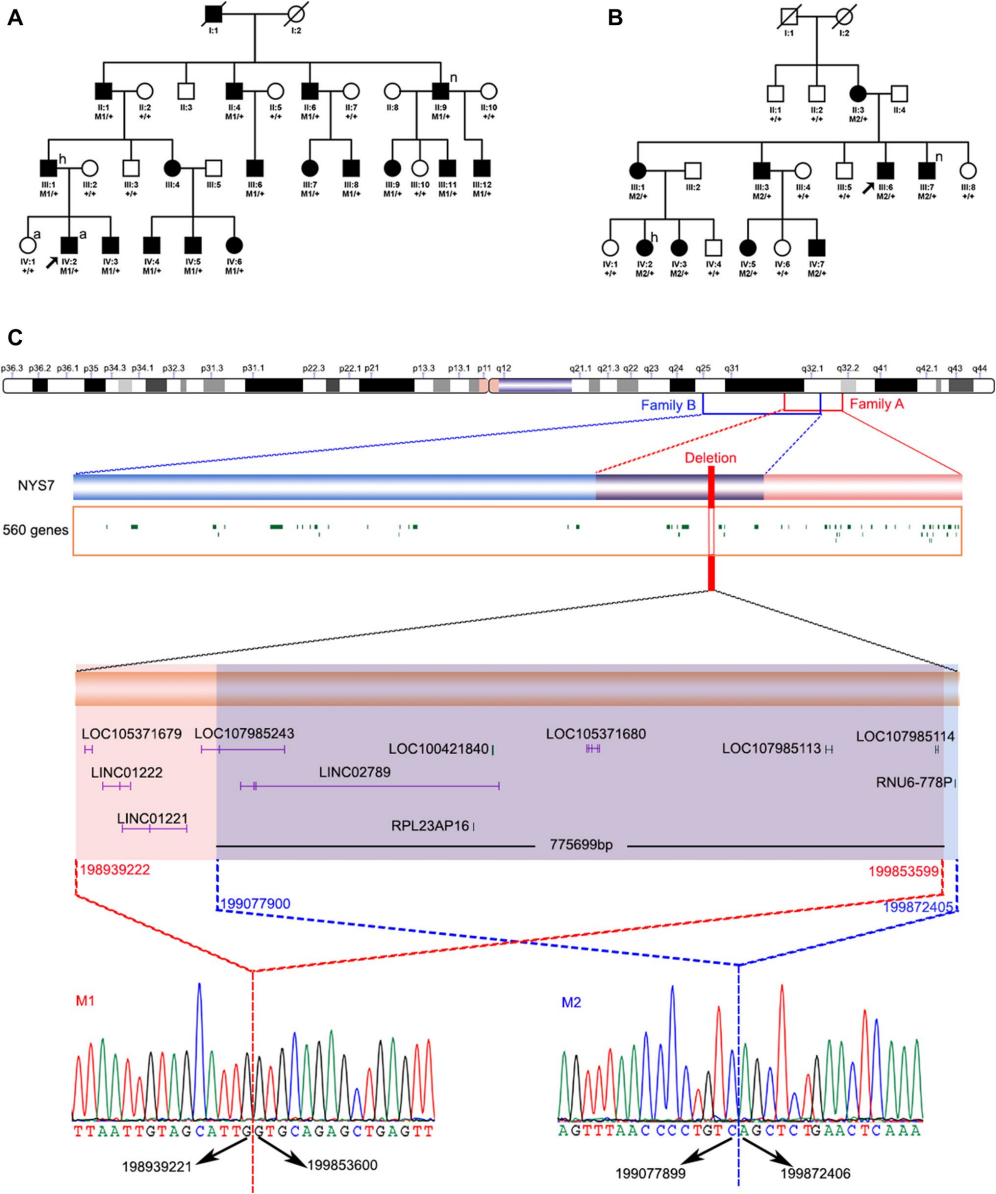
Two deletions were identified in the two families with CMN. **a** Pedigree of family A and genotypes of the M1 deletion in each available family member. **b** Pedigree of family B and genotypes of the M2 deletion in each available family member. **c** Schematic map and sequences of the two SVs. The map shows that the two deletions on chromosome 1q32.1 are 0.9 Mb and 0.7 Mb in size, respectively. The two squares just below the chromosome show the linkage intervals in the two families. Sequences of the breakpoints by Sanger sequencing are shown at the bottom. The six genes encoding an ncRNA (blue bar) and five pseudogenes (green bar) in the region of overlap between the two deletions are shown above the sequences. M1, 198939222–199853599 on chromosome 1 based on hg19. M2, 199077900–199872405 on chromosome 1 based on hg19

A total of forty-one individuals from two families with CMN were recruited for this study, including 25 affected individuals and 16 unaffected individuals as described in our previous studies (Fig. 1 a and b). Written informed consent was obtained from all individuals or their guardians prior to the study according to the tenets of the Declaration of Helsinki and following the Guidance of Sample Collection of Human Genetic Diseases (863-Plan) by the Ministry of Public Health of China. The diagnostic criteria of CMN, the preparation of genomic DNA from peripheral blood obtained from all participants, and the genome-wide linkage analysis have been described in our previous studies (Li 2012; Xiao et al. 2012). CMN in both of the families has been mapped to 1q31.3–q32.1 by genome-wide linkage analysis but no potential pathogenic variants have been identified by WES or candidate gene sequencing.

### Whole-genome sequencing by short-read sequencing platform

Whole-genome sequencing was performed using genomic DNA from peripheral blood obtained from two affected members, i.e., III:1 from family A and IV:2 from family B (Fig. 1), using the HiSeq2000 short-read sequencing platform (Illumina, San Diego, CA). The library was prepared using a TruSeq DNA Sample Preparation Kit (Illumina, San Diego, CA) according to the manufacturer’s instructions. In brief, after the genomic DNA was fragmented, end repair and A-tailing were performed. A hybridization reaction was performed to ligate the indexing adapters to the fragments and the products were purified to select fragments with length of 300–400 bp. Finally, these fragments were enriched using PCR. The library was qualified and then sequenced on the Illumina HiSeq genome analyzer platform (Illumina, San Diego, CA) with a mean coverage of 30-fold. The reads were aligned with the consensus sequence (UCSC hg19) for variant detection by the Burrows–Wheeler Aligner (BWA, https://bio-bwa.sourceforge.net/). Variants, including single-nucleotide variants (SNVs), small insertions and deletions (InDels), and structural variations (SVs), were filtered using GATK (https://gatk.broadinstitute.org/hc/en-us) and annotated using ANNOVAR (https://annovar.openbioinformatics.org/en/latest/).

### Whole-genome array-based comparative genomic hybridization analysis

Whole-genome array-based comparative genomic hybridization (aCGH) was performed on DNA samples of two members, i.e., IV:1 and IV:2 from family A, by a commercial service provided by Macrogen (https://www.macrogen.com/en/main/index.php) using the NimbleGen Human CGH 385 K Whole-Genome Array (Roche, Basel, Switzerland) with a capacity of 385,000 probes. In brief, genomic DNA from the affected member (IV:2) was considered the test DNA; while, genomic DNA from his unaffected sister (IV:1) was set as the reference DNA. The test sample (IV:2) was labeled with Cy3 dye and the reference sample (IV:1) was labeled with Cy5 dye. The labeled DNA was then hybridized with the NimbleGen CGH chip. Finally, the arrays were scanned using a GenePix4000B scanner (Axon Instruments, Foster City, CA, USA) and analyzed using the NimbleScan 2.5.

### Whole-genome sequencing by a long-read sequencing platform

Long-read genome sequencing was performed on two affected individuals with CMN from the two families, i.e., II:9 from family A and III:7 from family B (Fig. 1), by the Oxford Nanopore platform using a commercial service provided by GrandOmics (https://www.grandomics.com). The procedure for long-read genome sequencing has been previously described (Liu et al. 2019). In brief, size selection was performed on genomic DNA from the two affected individuals using Blue Pippin (cassette kit: BUF7510; size range: 30–40 kb). After assessment of DNA integrity and concentration, the library was prepared with a SQK-LSK108 library preparation kit and sequenced using PromethION sequencing. The data from PromethION sequencing were first analyzed using Guppy software for base calling. Then, SVs were detected by NGMLR-Sniffles and annotated by ANNOVAR. SVs were considered to be potentially pathogenic if they were absent from the Database of Genomic Variants (DGV, https://dgv.tcag.ca/dgv/app/home), and 1000 Genomes (https://phase3browser.1000genomes.org/index.html) databases.

### Sanger sequencing

Sanger sequencing was used to validate the potential pathogenic variants that were detected by the Oxford Nanopore platform and for segregation of candidate variants in the two families. Two pairs of primers were designed to validate the two SVs in the two families. The forward and reverse sequences of primers used for validation for the SV in family A were 5′-agaccatgggccaaagtgtt-3′ and 5′-agcatcagtagccgattcga-3′, respectively (M1-F and M1-R in Table S1). The forward and reverse sequences of primers used for validation for the SV in family B were 5′- gcttttctcctggtgctttca-3′ and 5′- accattgtactcccagcctg-3′, respectively (M2-F and M2-R in Table S1). An additional pair of primers (WT-F and WT-R in Table S1) was designed to validate hemizygosity of the region in the two families. The procedures that were used for amplification and sequencing of the targeted variants were previously described (Chen et al. 2013).

## Results

Potentially pathogenic variants for the two families were analyzed according to their respective linkage intervals based on whole-genome sequencing on HiSeq platform. No potential pathogenic SNVs or InDels were detected in either intronic sequence with predicted effect or coding region of the protein-coding genes located inside the two linkage intervals. However, two novel SVs were detected in the two families and these two SVs are partially overlapped. In affected individuals, all SNVs within the region of interest were homozygous, providing further support for the existence of a large deletion. An aCGH analysis further suggests a deletion of about 0.89 Mb inside the linkage interval in family A (Figure S1).

To clarify the boundaries of the deletion, long-read sequencing by the Nanopore platform was performed on samples from two affected members from the two families. Two SVs within the linkage interval were detected: a deletion of 914,377 bp in family A and a deletion of 794,505 bp in family B. Neither of the two SVs was present in the DGV or 1000 Genome database. The exact deletions inside the linkage interval for the two families with CMN were confirmed by Sanger sequencing of the fragments containing the breakpoints, i.e., chr1: 198939222_199853599del (hg19 genome assembly) in family A and chr1: 199077900_199872405del (hg19 genome assembly) in family B (Fig. 1). The two SVs partially overlapped and co-segregated with CMN in the two families (Figs. 1, S2).

**Fig. 2:**
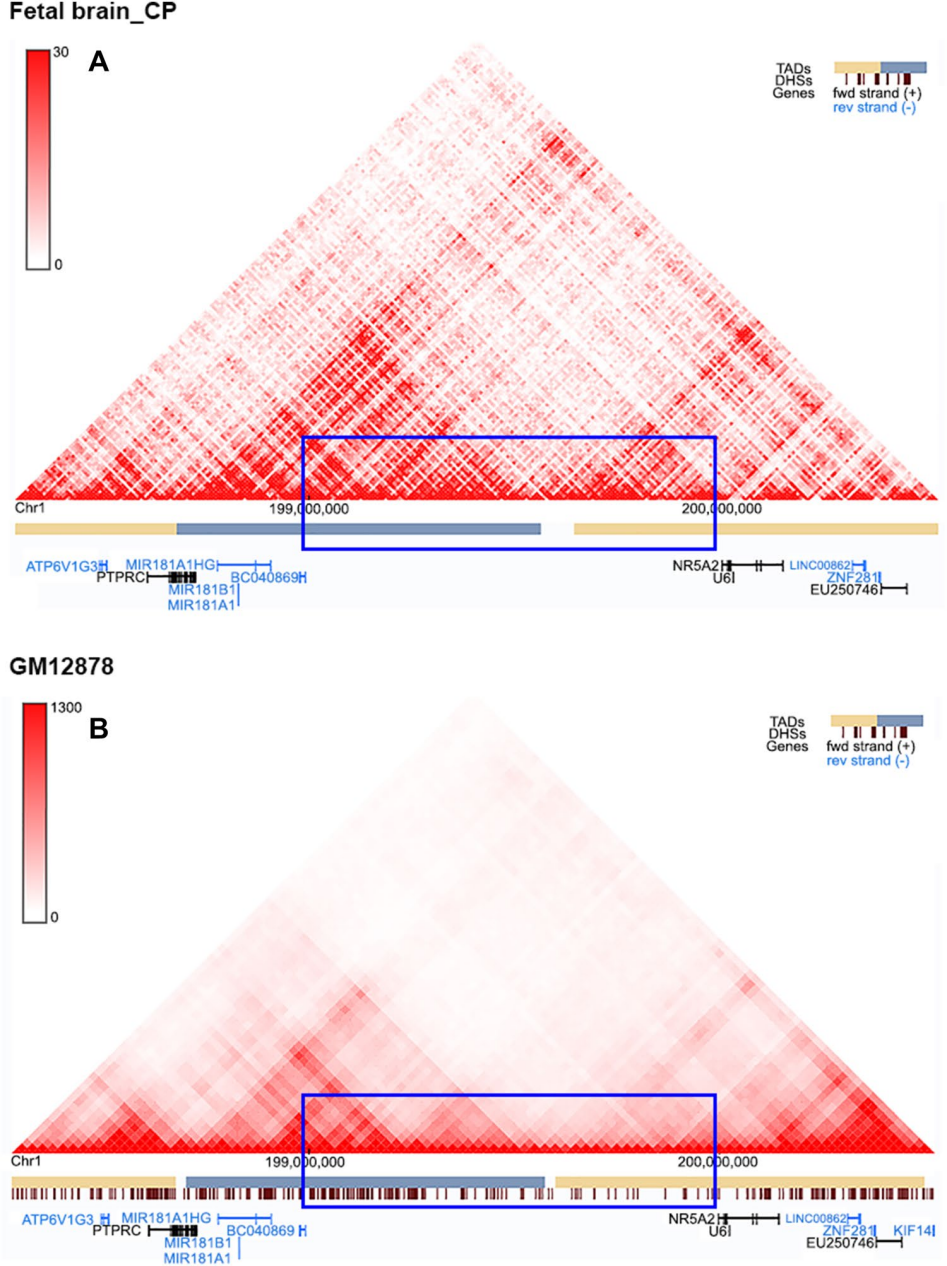
Schematic presentation of Hi-C maps of the overlapping deletion regions. The overlapping region was organized in two TADs according to Hi-C data based on both GM12878 (**a**) and fetal brain tissue (**b**). Only two genes, namely, *PTPRC* and *NR5A2*, are located at the two TADs

There are no protein-coding genes inside the deleted regions, just six uncharacterized non-protein-coding RNAs (ncRNAs) and five pseudogenes (Fig. 1, Table 1). Furthermore, analysis of the 3D genomic architecture based on TAD data around the deleted regions was obtained using the Hi-C 3D Genome Browser database from the Yue lab (https://promoter.bx.psu.edu/hi-c/index.html). The overlapping deleted region was organized into two TADs based on both GM12878 (a human lymphoblastoid cell line) and fetal brain tissue (Fig. 2). Therefore, the large deletions in the two families probably resulted in the formation of new TADs, disrupting the original boundary of the existing TADs. Only two gens, namely, *PTPRC* and *NR5A2*, are located within the two TADs.

**Table 1:** Uncharacterized genes and pseudogenes in the region of the two SVs

Gene symbol	Position	Gene type	Gene description	Expression
LOC105371679	198945304..198953041	NcRNA	Uncharacterized	Low expression in human tissues
LINC01222	198961718..198988093	NcRNA	Long intergenic non-protein-coding RNA 1222	Low expression in human tissues
LINC01221	198985262..199045864	NcRNA	Long intergenic non-protein-coding RNA 1221	Low expression in human tissues
LOC107985243	199068935..199148054	NcRNA	Uncharacterized	Low expression in human tissues
LINC02789	199117726..199362435	NcRNA	Long intergenic non-protein-coding RNA 2789	/
RPL23AP16	199340984..199341418	Pseudo	Ribosomal protein L23a pseudogene 16	/
LOC100421840	199356269..199357597	Pseudo	Eukaryotic translation elongation factor 1 alpha 2 pseudogene	/
LOC105371680	199418107..199429562	NcRNA	Uncharacterized	Low expression in human tissues
LOC107985113	199715844..199722018	Pseudo	2-iminobutanoate/2-iminopropanoate deaminase pseudogene	/
LOC107985114	199846106..199847532	Pseudo	Cytochrome c oxidase subunit NDUFA4 pseudogene	/
RNU6-778P	199857287..199857389	Pseudo	RNA, U6 small nuclear 778, pseudogene	/

A total of 106 different SVs have been reported overlapping with the two SVs identified in our families according to the DGV database and two regions common to the SVs in our families were not covered by any of the 106 SVs, that is the 199,407,000–199,436,000 and 199,631,000–199,641,000 regions (Figure S2). The former region involves an ncRNA coding gene (*LOC105371680*) that shows low expression in normal human tissues, including the human brain, according to the NCBI database (https://www.ncbi.nlm.nih.gov/gene/?term=LOC105371680); while, the latter region is located at the boundary of the two TADs. Seven pathogenic/likely pathogenic copy number variations were reported according to the DECIPHER database (https://decipher.sanger.ac.uk), all of which involved not only the deletion regions in the present study but also multiple protein-coding genes (Figure S3).

## Discussion

In this study, whole-genome sequencing based on a HiSeq platform excluded potential pathogenic mutations in genes inside the linkage interval. Two novel large deletions with a 775,699 bp overlap inside the linkage interval of NYS7 at 1q32.1 were identified by different platforms. Sanger sequencing clarified the breakpoints and confirmed the segregation of the deletions with CMN in the two families. The deleted regions had no protein-coding genes but were predicted to disrupt the 3D genome architecture in this region. These lines of evidence suggest that SVs of a chromosome region at 1q32.1 without any known protein-coding genes are responsible for CMN mapped to NYS7, for which structural variations due to deletions involving chromosome regions without any protein-coding gene have not been described.

Large SVs are the least studied among all kinds of variations for Mendelian diseases because of the difficulty in detection and clarification as well as the rarity of large families. Recently, a growing role of SVs has been reported in Mendelian diseases with the improvement of methodology development for sequencing and data analysis (Alkan et al. 2011; Sanchis-Juan et al. 2018), mostly based on analysis of regulatory elements inside or close to a known functional gene, such as the duplications downstream of *IRX1* for North Carolina macular dystrophy (Cipriani et al. 2017). The majority of disease-causing SVs are located in regions involving the coding regions of known genes associated with the diseases (Stankiewicz and Lupski 2010; Weischenfeldt et al. 2013). Several disease-causing SVs have been identified in non-coding regions, but these regions are either within intronic regions of known genes or in upstream or downstream regulatory elements of known genes (Benko et al. 2011; Klopocki et al. 2008; Lohan et al. 2014). However, the two SVs identified in the present study involve neither protein-coding genes nor the regulatory regions of known genes associated with CMN, i.e., *FRMD7* in the X chromosome. A recent study provides evidence that SVs could lead to misexpression of targeting genes by disrupting TADs (Diament and Tuller 2019; Lupianez et al. 2015), which is the first evidence that Mendelian diseases may be caused by disruption of 3D genome architecture due to SVs. In this study, the overlapping region of the two SVs was predicted to span the boundary of two TADs, in which the *PTPRC* and *NR5A2* genes were involved. Mutations in *PTPRC* have been identified in patients with autosomal recessive severe combined immunodeficiency (SCID) (Kung et al. 2000); while, pathogenic mutations in *NR5A2* have not been reported for any Mendelian disease. *PTPRC* is a member of the protein tyrosine phosphatase family and functions as regulator of a variety of cellular processes, including T- and B-cell antigen receptor signaling (Xu and Weiss 2002); while, *NR5A2* encodes a hepatocyte transcription factor and has been reported to play a role in immune cells (Seitz et al. 2019). Variants in the two genes were analyzed based on the WES data from 22 probands with CMN, in which mutations in *FRMD7* were excluded. No potential pathogenic variants in them were identified in the probands based on systemic analysis of our in-house WES data from 3280 individuals. Therefore, it is as yet unknown whether these two genes might contribute to CMN in other families, although both genes are expressed in the human brain according to information from GeneCards (https://www.genecards.org/). Another possibility is that the SVs could affect the expression of genes in a distal region (Lesne et al. 2014), even *FRMD7* on the X chromosome. Further studies will be required to definitively prove the causality of the two SVs by clarifying the mechanism of them underlying autosomal dominant CMN. Analysis of additional families with autosomal dominant CMN may further narrow down the critical region of the SVs. Disruption of TADs as well as their targeting gene regions might be further investigated by Hi-C analysis based on brain organoids from patient-specific induced pluripotent stem cells derived from patients of the two mapped families.

Although six ncRNA genes are involved in the two deletion regions, three of them are locate at the overlapping region of the two deletions. Moreover, only the uncharacterized ncRNA coding gene (*LOC105371680*) remained at the regions uncovered by the 106 reported SVs in the overlap of the two deletion regions. However, this ncRNA gene has a low expression in the human brain. Therefore, the association between the CMN phenotype and these ncRNA genes need to be investigated in additional families with CMN.

In conclusion, two novel SVs in non-coding region in 1q32.1 were identified in two large families with CMN in this study. The SVs are predicted to lead to changes in 3D genomic architecture by disrupting TADs. These results provide additional suggestive evidence of a strong association between SVs in non-coding genomic regions and human hereditary disease, possibly by changing the 3D genome architecture, which provides clues regarding the molecular pathogenicity of CMN. To our knowledge, this is the first study on pathogenic SVs in non-coding genomic regions without any related protein-coding genes in CMN.

## Electronic supplementary material

Below is the link to the electronic supplementary material.

Supplementary file1 (XLSX 9 kb)

Supplementary file2 Supplementary Figure 1. The log2 ratio plots of chromosome 1 in IV:2 from family A by aCGH. An approximately 0.89 Mb deletion was detected at chr1:197217711-198112096 based on GRCh37/hg19 (blue square). (TIF 1166 kb)

Supplementary file3 Supplementary Figure 2. The SVs reported in DGV in the deletion interval of the two families. Two regions were not covered by any of these SVs, that is 199,407,000–199,436,000 and 199,631,000–199,641,000 regions, which have been indicated by red squares. (TIF 2295 kb)

Supplementary file4 Supplementary Figure 3. SVs reported in DECIPHER database in the deletion interval of the two families. Seven pathogenic/likely pathogenic copy number variations were reported, all of which involved not only the deletion regions in the present study but also multiple protein-coding genes. (TIF 5634 kb)
