# Supplementary figures and images for: Structural variations in a non-coding region at 1q32.1 are responsible for the NYS7 locus in two large families
Source: Hum Genet. 2020 Apr 4;139(8):1057–64. doi: 10.1007/s00439-020-02156-0 (PMC7406531; doi:10.1007/s00439-020-02156-0)

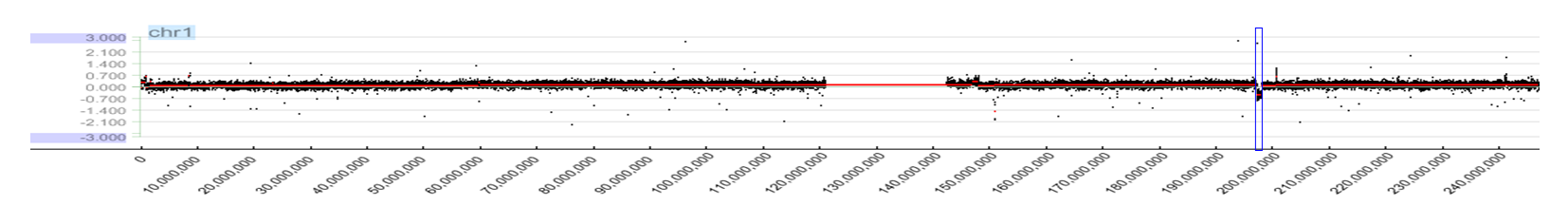

Supplement: Supplementary file 2 — Supplementary file2 Supplementary Figure 1. The log2 ratio plots of chromosome 1 in IV:2 from family A by aCGH. An approximately 0.89 Mb deletion was detected at chr1:197217711-198112096 based on GRCh37/hg19 (blue square). (TIF 1166 kb) [file 439_2020_2156_MOESM2_ESM.tif]

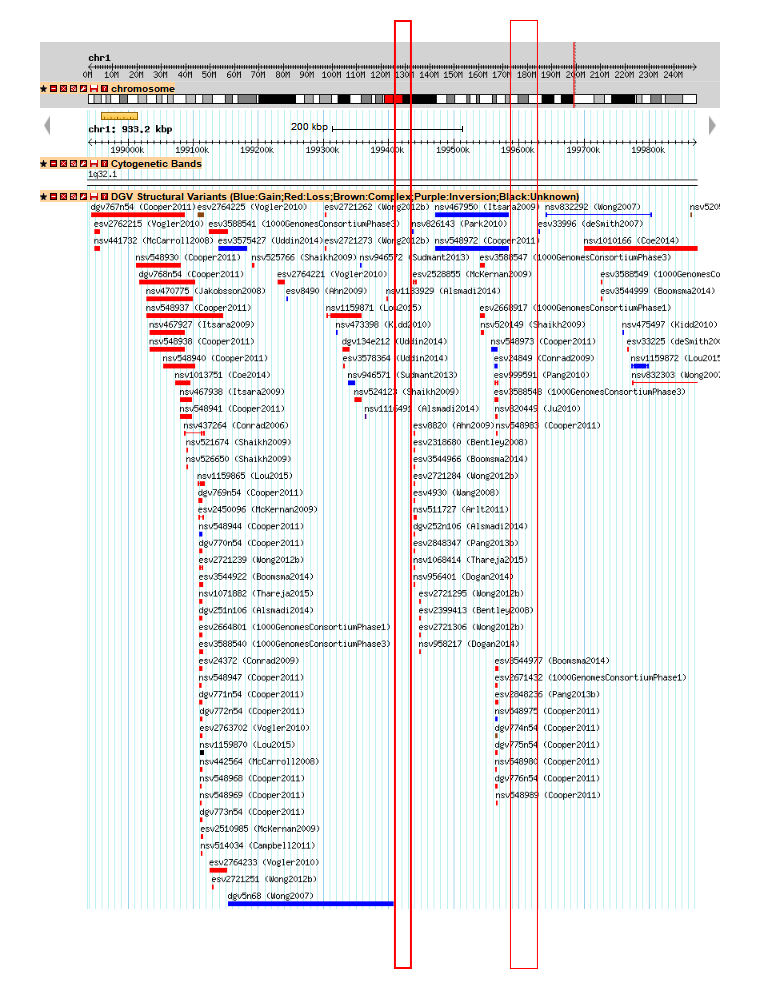

Supplement: Supplementary file 3 — Supplementary file3 Supplementary Figure 2. The SVs reported in DGV in the deletion interval of the two families. Two regions were not covered by any of these SVs, that is 199,407,000–199,436,000 and 199,631,000–199,641,000 regions, which have been indicated by red squares. (TIF 2295 kb) [file 439_2020_2156_MOESM3_ESM.tif]

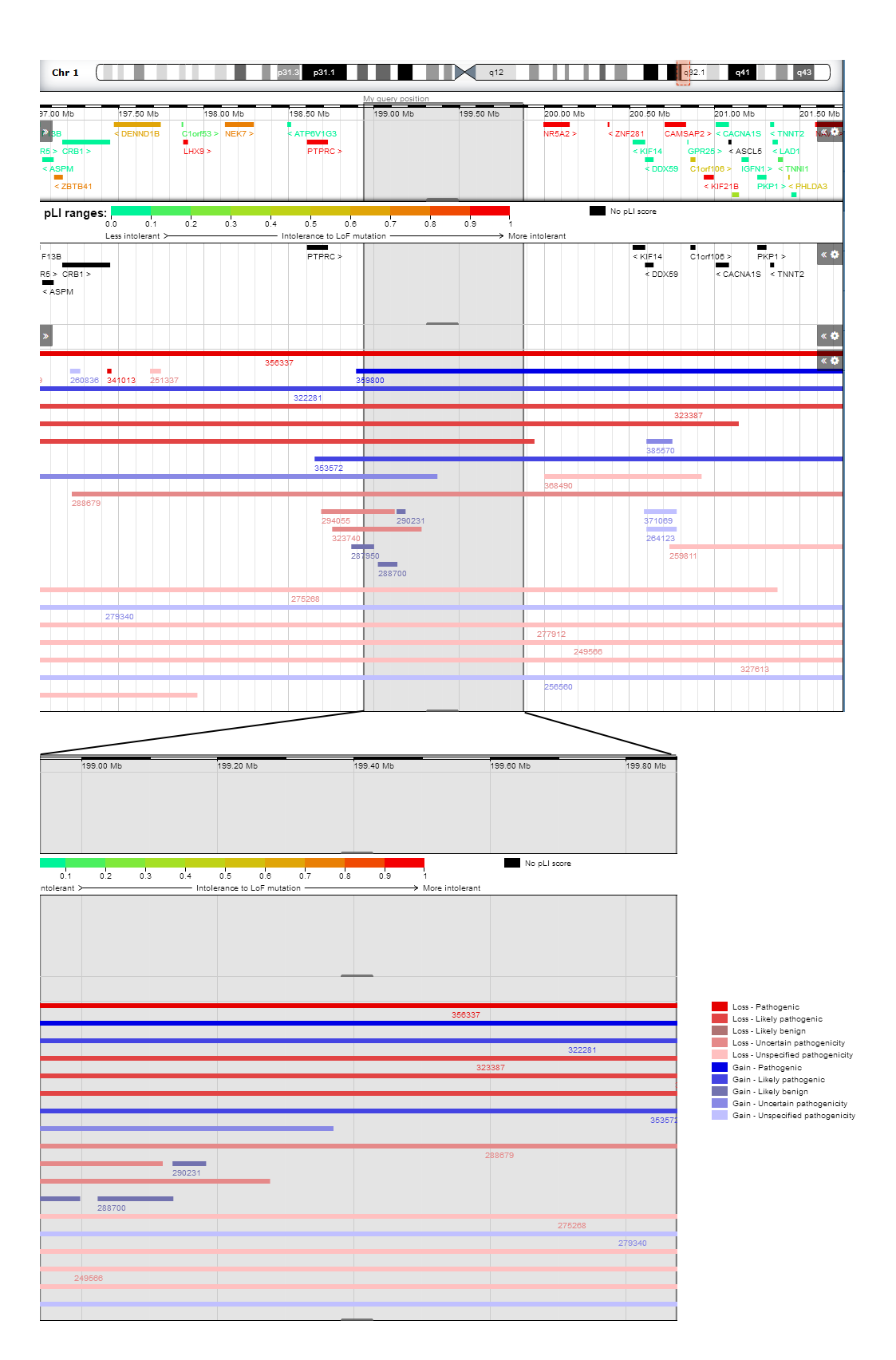

Supplement: Supplementary file 4 — Supplementary file4 Supplementary Figure 3. SVs reported in DECIPHER database in the deletion interval of the two families. Seven pathogenic/likely pathogenic copy number variations were reported, all of which involved not only the deletion regions in the present study but also multiple protein-coding genes. (TIF 5634 kb) [file 439_2020_2156_MOESM4_ESM.tif]
